# Collectanea Medica

**Published:** 1813-07

**Authors:** 


### COLLECTANEA MEDICA,

CONSISTING OF

ANECDOTES, FACTS, EXTRACTS, ILLUSTRATIONS, QUERIES, SUGGESTIONS, &c.

RELATING TO THE

*History or the Art of Medicine, and the Auxiliary Sciences.*

---

### *Analysis of the Cerebral Matter of Man, and some other Animals. By* M. Vauquelin.

Sect. I.—*History of the chemical labors hitherto undertaken on the cerebral matter.*

ALTHOUGH the brain, in consequence of the functions which it is supposed to perform, ought to have early excited the curiosity of chemists, yet one is surprised to find but very little in their works concerning its chemical nature. Even the small number of experiments which have been undertaken, have not been pushed far enough to enable us to deduce any positive consequences. Hence the opinions formed respecting the composition of the brain are erroneous, or at least incomplete. It was therefore necessary to resume the subject from the commencement, and to employ that care and precision which the difficulty of the subject rendered necessary. I have undertaken this difficult task. I submit the results which I have obtained to the chemists. It is their province to judge how far I have succeeded.

Gurman first announced the long period during which the brain remains sound in the cranium of dead bodies.

Burrhus compared this organ to an oil, and particularly to spermaceti.

Thouret, whose loss Medicine laments, in an excellent memoir on the dead bodies found in the burying-ground of the Innocents, considered the substance of the brain as a sort of soap.

---

Fourcroy,

Fourcroy, whom the Sciences likewise deplore, advanced an opinion respecting the nature of the cerebral matter different from that of Thouret.* He considered it as principally composed of albumen and of another matter, which he thought a peculiar substance. Though the experiments of Fourcroy leave several things imperfect, yet it will be seen, by comparing them with mine, that his account of the brain is by far the completest hitherto given, and that it approaches pretty closely to the truth.

SECT. II.—*Treatment of the brain with alcohol, or spirit of wine.*

A portion of human brain, deprived of its envelopes, and reduced to a homogeneous pulp in a marble mortar by means of a wooden pestle, was mixed with about five times its weight of alcohol of 36 degrees. This mixture, left to macerate during 24 hours, was heated to the boiling temperature, and passed through the filter.

The alcohol had acquired a greenish color. It deposited on cooling, a white matter, partly in flocks, and partly in plates.

Twelve hours after the cooling, the alcohol was filtered again. It still retained its green color. Water destroyed its transparency, and rendered it milky.

This alcohol, being evaporated till only one-eighth part of it remained, deposited, on cooling, an oily matter, yellowish and fluid, which sunk to the bottom of the vessel. The liquid itself continued yellowish.

We shall hereafter examine this oily matter, together with the liquor which accompanied it.

The alcohol obtained by distillation was poured upon the cerebral matter, already once digested with alcohol, as has been already said.

After having boiled the mixture for a quarter of an hour, the alcohol was filtered while hot. It passed through the filter with a color approaching to blue, and deposited, on cooling, a white matter, as in the first operation, but less abundant. The alcohol, after having deposited this matter, still became milky when mixed with water. This alcohol, when distilled, passed without color; and the residue of the distillation, which amounted to about the 28th part of the liquid subjected to distillation, had lost its green color, and acquired a yellow color.

This residue exhibited two sorts of liquors: one of which had the aspect of an oil, and occupied the bottom of the vessel; the other, less colored, resembled a solution of gum.

We defer the examination of these two liquids till we come to describe those which were obtained by the first operation, because we suspect them to be of the same nature.

The white matter deposited by alcohol in the first operation, and that which the same liquid allowed to deposite in the second operation, had a pasty consistence, a greasy and glutinous feel, a brilliant and satiny appearance.

---

* Annales de Chimie, vol. xvi.

The

The last portion was whiter and more solid; but being melted, it was changed, like the first, upon being brought near the flame of a candle.

These substances, when dried upon filtering paper, rendered it transparent, and stained it as an oil would have done.

The matter, which had been retained in solution by the alcohol, and which had been separated by the distillation of this liquid, had a yellow color, and was of the consistence of a paste, and adhesive. When dried, it dissolved again in boiling alcohol; but before entering into combination with the liquid, it melted at the bottom of the vessel, and assumed the appearance of an oil. The alcoholic solution deposites, on cooling, two matters, which probably differ from each other in the aspect only: the one, which precipitates first, attaches itself to the sides of the vessel under the form of a yellow, thick, tenaceous fat; the other remains suspended in the liquor, under the form of scales, white and brilliant, like boracic acid.

SECT. III.—*Desiccation of the brain.*

Nine ounces, one gros (about 292 grammes, or 4312 grains troy, or very nearly three quarters of a troy pound), of cerebral matter, when dried over the water-bath, were reduced to two ounces, or nearly to a fifth part of their original weight; but the desiccation was not complete. These two ounces of matter, burnt in a platinum crucible, decrepitated and melted, and produced a smoke, which had the odor of an empyreumatic oil. This oil, in burning, gave a yellowish white and very large flame, and deposited a great deal of lamp-black. Then the odor of the empyreumatic oil became imperceptible. As soon as the flame ceased, the crucible was withdrawn from the fire. The charcoal which it contained weighed $5\frac{1}{10}$ grammes (1 gros, 25 grains; or 78·7 grains troy). It was reduced to powder, and exposed again to heat in a platinum crucible. Though exposed to a violent heat, it did not appear to burn; but softened, assuming a pasty form.

After having been exposed for an hour to a white heat, its weight was still 4·68 grammes ($72\frac{1}{4}$ grains troy); so that it had only lost 38 hundred parts of a gramme, which demonstrates a very difficult combustion in this charcoal.

Being washed with boiling water, and dried, it now weighed only 2·36 grammes (36·5 grains troy). Hence it had lost 2·32 grammes.

The solution strongly reddened the tincture of litmus; and the precipitate which lime-water formed in it was re-dissolved, till the excess of acid was saturated.

The same charcoal, exposed to heat a second time, burnt with a slight flame of phosphorus; but after a certain interval it softened as before, and assumed the form of a paste. It was washed a second time, and the water became acid, as before. These processes were repeated in the same manner till the whole of the charcoal was consumed.

The water employed in washing the charcoal being evaporated, yielded a white deposite, with a tint of blue, and a pasty consistence. This

This deposite, being separated from the liquor by the filter, melted very readily into a transparent glass. The same deposite reduced to powder, and mixed with diluted sulphuric acid, furnished sulphate of lime, but in a quantity which did not correspond with that of the matter employed.

Ammonia being mixed with a small portion of the liquid from which the above-mentioned deposite had been separated, occasioned only a very slight precipitation. Caustic potash, on the contrary, occasioned a very plentiful one. This last precipitate was chiefly magnesia, while the deposite formed spontaneously in the liquor was phosphate of lime.

As every thing seemed to show that the acidity of the liquor mentioned above was due to phosphoric acid, lime-water was mixed with it till no farther precipitation took place. This last precipitate being washed, was dissolved in muriatic acid, and the lime precipitated from it by means of oxalate of ammonia. The liquor of this last experiment was treated with caustic potash; but no precipitate took place while it remained cold. A boiling heat being employed, a flocky precipitate was obtained, which possessed the properties of magnesia.

The liquor precipitated by lime-water, as mentioned above, was evaporated in an open vessel, that the excess of lime might fall down. After filtration this liquid had a yellowish color, a caustic taste, and precipitated abundantly muriate of platinum yellow. This liquor, when concentrated, was left in the open air, that it might crystallize, and that it might be seen whether it contained soda; but all the experiments to which it was subjected demonstrated that it was only potash partly saturated with carbonic acid.

These experiments on the combustion of the brain prove that the salts contained in that organ are phosphates of lime, of magnesia, and of potash.

The matter of the brain, after having been repeatedly boiled in alcohol, being burnt in a platinum crucible, exhibited almost the same phenomena as the brain in its natural state; that is to say, it decrepitated and flamed, but emitted less smoke; and its charcoal being calcined, did not soften, and gave no signs of acidity. This proves that the constituents which produced this effect in the entire brain were removed by the alcohol. We shall see hereafter what these constituents are.

SECT. IV.—*Examination of the fatty matter of the brain which is deposited during the cooling of the alcohol in which brain has been boiled.*

We have already described the principal physical characters of this substance: we have said that it was white and solid, but soft, and of a pitchy consistence; that it had a brilliant and satin-like aspect; and that it stained paper in the same manner as oils do. We shall now examine its chemical nature and composition.

1. When exposed to heat it melts, but it does not become so fluid as tallow does, and assumes a brown color at a temperature at which common fat is not altered.

2. It dissolves in hot alcohol, leaving only a few flocks of animal matter, which had been dissolved in the first operation by means of the water contained in the brain. During the cooling of the alcohol the greatest part of this matter precipitates with all its usual characters: 20 parts of alcohol at 36 degrees are sufficient to dissolve one part of this matter.

3. When exposed to the sun this matter acquires a yellow color, nearly similar to that of the fatty matter which is obtained by the evaporation of the alcohol, after it has deposited the fatty matter, the properties of which we are describing. I do not know the reason of this phenomenon.

4. A portion of this matter, which had been dissolved several times in alcohol to separate the last remains of the animal matter which it contained, was burnt in a platinum crucible. The combustion took place very readily, and was accompanied by a great deal of flame and smoke. The charry residue washed with distilled water communicated to that fluid a very distinct acidity, and the property of precipitating lime-water.

The singular result of this operation, which announced unambiguously the presence of phosphoric acid, made me suspect that this fatty matter contained phosphoric acid, or phosphate of ammonia, the base of which might have been volatilized by heat, though this last opinion was not very probable. However, to determine the point, I made the following experiments:—

1. I mixed the fatty matter with distilled water, and observed with surprise that it formed with that fluid a kind of emulsion, and did not separate from it. At the same time, I observed that this emulsion possessed no acid properties, and did not alter the color of tincture of litmus.

2. I mixed it with a solution of caustic potash, and perceived no indication of the presence of ammonia. Even a boiling heat did not develope the smallest trace of this alkali. In this experiment I was very much surprised to perceive, that though I had employed a quantity of potash more than sufficient to dissolve a quantity of tallow, more than I employed, yet the solution did not take place; and the mixture remained as milky as if water had been employed instead of potash.

I think we may conclude from these experiments that the fatty matter from the brain contained neither phosphoric acid nor phosphate of ammonia, and that the acid which appears after the combustion had another origin.

3. A hundred parts of the fatty matter of the brain were heated in a platinum crucible, with 200 parts of potash and a little water. The mixture did not melt; but, on the contrary, became harder, which would not have happened if the substance in question had been real tallow. When the humidity was dissipated it assumed a brown color, took fire, emitted an odor of burning grease, and gave out a great deal of smoke. The residue of this operation was washed with distilled water; the liquid being saturated with nitric acid, and boiled, gave, when mixed with lime-water, a flocky precipitate, which was phosphate of lime, and which weighed, when dry, the tenth part of the mass employed.

4. A hundred parts of the same matter thrown successively into melted nitre took fire with great facility, producing scarcely any smoke; the whole was destroyed, and not the smallest trace of charry matter remained. The residue of this operation, treated in the same manner as the preceding, gave the same quantity of phosphate of lime.

What conclusion can be drawn from these experiments, except that there is phosphorus combined with the fatty matter of the brain, and which dissolved in alcohol at the same time with the fatty matter? We find in the residue after combustion neither phosphate of lime nor phosphate of magnesia. The alkaline phosphates would have found enough of water in the brain to remain in solution in the alcohol, and not to precipitate when the liquid cools. Accordingly we find phosphate of potash, superphosphate of lime and of magnesia, in the residue of the alcohol evaporated, which had been digested with the cerebral matter. We must therefore admit the existence of phosphorus in the brain, as well as in the roes of fishes, where it was discovered by Fourcroy and me. The proportion of it, indeed, is very small; for, from the quantity of phosphate of lime which I obtained in the preceding experiments, I estimate its quantity not to exceed $\frac{5}{100}$ths of a part: but if we subtract the humidity of the brain, and only consider the dry residuum, in that case the phosphorus may be considered as amounting to about $\frac{1}{100}$th part of the whole.

Though the substance whose properties have been described in this section has more analogy with tallow and fat than with any other class of bodies, yet it ought not to be confounded with ordinary fat. It differs from it principally by its solubility in alcohol, its capacity of crystallizing, its viscidity, its inferior fusibility, and the black color which it assumes in melting. Thus, though we class it among fatty bodies, we ought to consider it as a particular and new species.

### SECT. V.—*Of the fatty matter of the brain which remains in solution in the alcohol after its cooling.*

We have observed before, that after the alcohol digested with the brain had deposited its fatty matter, it remained of a green color; and that the third, the fourth, and even the fifth, portion of alcohol, which had been digested on the same portion of brain, had a sapphire-blue color. In order to discover the coloring matter, we distilled this alcohol. The following are the observations that we made:—

The green and blue color is not destroyed by the evaporation of the alcohol, as long as any of the alcohol remains; but as soon as the whole is driven off, the matter acquires a yellow color, of more or less intensity. Neither the alkalies nor acids change these colors.

When these operations are performed on the first and second portions of alcohol which have been digested on the same quantity of brain, brain, we see, as has been mentioned above, an oily fluid of a yellow color precipitate itself to the bottom of the aqueous fluid derived from the humidity of the brain. But this effect does not take place with the last portions of alcohol, because they contain no more water.

The liquid, at the bottom of which this fatty matter collects, has likewise a yellow color, a taste of the juice of meat, and slightly sweetish, and it gives marks of acidity. While this liquor is hot, the matter remains quite distinct, and seems to have some consistence; but by cooling, or on the addition of a little water, it absorbs humidity, becomes opake, and so mixed with the water that it cannot be separated. We must therefore take advantage of the favorable moment to make this separation in the proper manner.

From these remarks it is obvious that hot water must be employed to wash this substance, and to free it from the soluble matters with which it is mixed.

To dry this oil after washing it, we may expose it for some time to the open air, or to a gentle heat.

Let us examine the properties of this matter thus purified, leaving to another section the examination of the water from which it has been separated.

1. It has a reddish brown color, an odor similar to that of the brain itself, but stronger. Hence it is probably this substance which gives its peculiar odor to the brain.

2. Its taste is similar to that of rancid fat.

3. When agitated with cold water it mixes with that liquid, and forms a sort of homogeneous emulsion, which separates only very slowly. The mineral acids, mixed in a certain quantity with this emulsion, immediately precipitate an oily matter, under the form of white opake flocks; and the liquor then passes clear through the filter, which was not the case before. The muriatic acid which has thus served to coagulate this species of emulsion, lets fall very light white flocks, when mixed with ammonia; but when nitric acid is employed, it neither can be made to precipitate by ammonia nor lime-water.

The infusion of nutgalls likewise coagulates this emulsion.

4. If the water be decanted off as soon as the fatty matter is deposited, and it be left to itself, it putrifies, and exhales a fetid odor, indicating the presence of an animal matter.

5. It dissolves in hot alcohol, some light flocks excepted, which do not amount to the hundredth part of it. The greatest part of it separates from the alcohol when it cools, and renders it milky, as would happen to a solution of resin.

6. Exposed on burning coals it melts, blackens, swells up, and emits an odor of burning animal matter, and afterwards that of grease in the state of vapor.

7. When burnt in a platinum crucible, either alone or mixed with potash or nitrate of potash, it always furnishes phosphoric acid, either uncombined or combined with the alkali, according to the process; just as happens to the fatty matter deposited from the alcohol during its its cooling. Hence we must form the same opinion respecting the origin of this acid. We must admit the presence of phosphorus in the fatty matter.

From 400 grammes of brain employed in this process we have obtained about 3 grammes of this matter, which amounts to about 0 75 of a gramme per cent.

We ought now to inquire in what this substance differs from that which falls spontaneously from the alcohol during its cooling, the properties of which have been already described.

Though it remains in solution in the cold alcohol, it is not very soluble in that liquid; for when alcohol, at a boiling temperature, is saturated with it, a portion is deposited, as the alcohol cools, in the form of flocks. In this respect it approaches very near the first substance. It differs from it by its reddish brown color, by its smaller consistence, by its slight taste of boiled meat which the first substance has not, and by a greater tendency to crystallization.

This difference is produced by a certain quantity of animal matter, of which we shall speak hereafter, and which may be separated from the fatty matter by means of cold alcohol.

SECT. VI.—*Of the yellow aqueous liquor which remains after the separation of the two fatty substances, by cooling and by evaporating the alcohol.*

When one has deprived the brain, by means of alcohol, of every thing soluble in that liquid, and separated, by the methods above described, the two fatty substances from the alcohol, there remains a liquor of a brownish yellow color, which has the taste of the juice of meat with a little sweetness. This liquid reddens litmus; and is precipitated by lime-water, infusion of nutgalls, &c.

To learn the nature of the substances contained in that liquid, we in the first place diluted it with a quantity of distilled water, and then poured into it lime-water as long as any precipitate continued to fall. The matter washed and dried in the open air had a yellow color. When calcined it assumed a black color, owing to the presence of a little animal matter, which is decomposed by the heat.

This substance thus calcined and re-dissolved in nitric acid was again precipitated white by ammonia. It was not blackened by exposure to heat, and possessed the characters of phosphate of lime.

After having precipitated, by means of lime, the phosphoric acid contained in the aqueous liquid, we evaporated it to dryness with the requisite precautions. The matter which it furnished weighed 4·5 grammes (69·5 grains troy). In this state it had a reddish brown color, was semi-transparent, had a taste similar to the juice of meat with a little sweetness; it dissolved in alcohol with great facility, leaving only some atoms of a saline matter which effervesced with acids.

Exposed to the air it became soft by attracting humidity. A portion of this matter being heated in a platinum crucible, swelled up considerably, and emitted vapors which had the odor of burning animal matter. It left a charcoal, which yielded, when washed with with water and the liquid was evaporated, a little pure carbonate of potash.

It follows, evidently, from these experiments, that the aqueous liquid contained uncombined phosphoric acid and phosphate of potash, or perhaps superphosphate of potash and an animal matter, which by its solubility in alcohol and water, by its property of being precipitated by infusion of nutgalls, by its reddish brown color, its deliquescence, its taste and smell of the juice of meat, ought to be regarded as identical with the substance which Rouelle formerly called the saponaceous extract of meat, and to which M. Thenard has given the name of *osmazome*.

It is, without doubt, this substance, a portion of which remains with the fatty matter obtained from the alcohol by evaporation, which gives it the reddish color, the property of mixing with water, and of emitting the smell of animal matter when burning.

### SECT. VII.—*Statement of the constituents of the brain soluble in alcohol.*

We now know the different substances separated from the brain by alcohol when repeatedly digested upon it. They are,

1. A fatty matter, white, solid, of a satin lustre, and a tenacity not to be found in ordinary tallow or fat.
2. Another fatty matter of a red color, having less consistence than the preceding, but which seems to differ from it only in consequence of a little osmazome which remains mixed with it.
3. An animal matter of a reddish brown color, soluble in water and alcohol, forming with tannin an insoluble combination, having the smell and taste of the juice of meat, and which is certainly the principle at present distinguished by the name of *osmazome*.
4. Superphosphate of potash, together with some traces of common salt, of which I have not spoken, because it occurs in all the animal humors.

### SECT. VIII.—*Examination of the part of the brain which is insoluble in alcohol.*

When we have separated, by repeated digestions in boiling alcohol, every part of the brain soluble in that liquid, there remains a greyish white matter in the form of flocks, which has the appearance of fresh cheese, but differs from that substance by its chemical properties: 400 grammes of fresh brain furnished 31 grammes of this substance.

This substance, in drying, assumes a grey color, a semitransparence, and a fracture similar to that of gum arabic.

Put into water in that state, it absorbs a portion of it, becomes opake, swells up, and softens. The water dissolves a small portion of it, for it becomes putrid after an interval of some days.

Thus softened, it dissolves readily by the assistance of heat in caustic potash, and during the solution no ammonia is disengaged, as is the case with the curdy portion of milk when dissolved in the same manner.

The

The solution of this substance in potash is slightly brown, its smell is not strong, the acids precipitate it in the form of white flocks, and disengage a very fetid odor. When acetate of lead is dropped into the solution, a dark brown precipitate falls, showing obviously the presence of sulphur.

Five grammes (77·2 grains troy) of this matter cautiously distilled, furnished carbonate of ammonia in crystals, and a red oil having a smell similar to that of albumen decomposed in the same manner. There remained in the retort 1 gramme (15·4 grains troy) of charcoal, which required 5 grammes of nitre to be entirely burnt.

The solution of salt obtained from it left 5 centigrammes (0·77 of a grain troy) of earthy residue, which was phosphate of lime. The liquid being supersaturated by nitric acid, and subjected to ebullition, let fall no precipitate; but it yielded a copious one when mixed with lime-water. This shows that the phoshate of magnesia had been decomposed by the potash, and perhaps even a portion of the phosphate of lime.

This matter, heated alone in a crucible, decrepitates, swells, and melts like albumen. Its charcoal, though calcined for a long time, does not become acid like that of the fatty matter; which shows that it contains no phosphorus. This charcoal, being washed with muriatic acid, furnished a small quantity of phosphate of lime and phosphate of magnesia.

When thrown into melted nitre it burns rapidly, and with flame; and we find, in the alkali resulting from that operation, very sensible traces of sulphuric acid, though the saltpetre employed contained none of it. This proves that the matter of the brain which is insoluble in alcohol contains sulphur; and confirms what was indicated by the acetate of lead dropped into the alkaline solution of this substance.

The properties which the portion of the brain insoluble in alcohol has presented, leave no doubt that it is perfectly identical with albumen. The knowledge of this circumstance explains very well the coagulation of the brain mixed with water by heat, acids, metallic salts, &c. This was the opinion which Fourcroy had formed of this substance in his memoir on the subject published in the Annales de Chimie.

### SECT. IX.—*General Result.*

The mass of the brain, then, is composed of the following substances:—

1. Two fatty matters, which are probably identical.
2. Albumen.
3. Osmazome.
4. Different salts; and among others, phosphates of potash, lime, and magnesia; and a little common salt.
5. Phosphorus.
6. Sulphur.

I conceive, as far as it is possible to draw conclusions from experiments periments so delicate, that these substances exist in the brain in the following proportions:—

| | | |
|---|---|---|
| 1. | Water | 80·00 |
| 2. | White fatty matter | 4·53 |
| 3. | Reddish fatty matter | 0·70 |
| 4. | Albumen | 7·00 |
| 5. | Osmazome | 1·12 |
| 6. | Phosphorus | 1·50 |
| 7. | Acids, salts, and sulphur | 5·15 |
| | | 100·00 |

SECT. X.—*Putrefaction of the cerebral matter.*

A portion of brain being diluted with a certain quantity of water, and abandoned to itself during a month, presented the following phenomena. At first it separated into three parts. That portion which occupied the surface was a part of the matter of the brain elevated by air bubbles attached to it. The portion in the middle was a yellow colored liquid, which, after an interval of some days, assumed a fine red color, which it retained for more than twenty days. After that period, this color by degrees faded, and was succeeded by a yellow color, more intense than that which the liquid had formerly possessed. The third portion, occupying the bottom of the vessel, was another part of the matter of the brain. During the month that it was allowed to remain, it emitted no gaseous matter.

The vessel containing this mixture being left open, there issued out of it an invisible vapor, having a disagreeable smell, somewhat similar to that of putrid cheese. Some persons compared it to the odor of the intestines when beginning to decompose.

A paper dipped in the solution of acetate of lead being exposed to this vapor, assumed immediately a blackish brown color.

The liquid in which the brain had thus putrified was sensibly alkaline; at least it restored the color of litmus paper reddened by acids, and formed white vapors when oxymuriatic acid was brought into its neighbourhood.

The liquid separated by filtration from the matter of the brain had an amber color. Acids rendered it muddy, throwing down white flocks. The odor which it exhaled in these circumstances was more fetid and disagreeable than before. Oxymuriatic acid rendered it muddy, likewise; but at the same time entirely destroyed its odor.

After being filtered, the liquor was subjected to distillation. As soon as it was heated nearly to the boiling temperature, yellow flocks separated in abundance, as happens when a diluted solution of albumen is treated in the same manner.

The product of the distillation was without color. Its odor was perfectly similar to that of the liquid before distillation. It precipitated acetate of lead white, and restored the color of litmus red-

 dened dened by an acid. Oxymuriatic acid destroyed its odor, and made it assume a yellow color.

When the liquor remaining in the retort was reduced to about a fifth part, it was filtered. Its color was yellow, and its odor similar to that of old cheese. It had become acid, for it reddened the color of litmus paper. Infusion of nutgalls, lime-water, and alcohol, formed flocky precipitates in it. Ammonia also occasioned a granular and semitransparent precipitate, which resembled ammoniaco-phosphate of magnesia. Concentrated sulphuric acid being mixed with this liquid developed a strong smell of vinegar.

The solid matter of the brain which had undergone fermentation, being washed with water, and submitted to the action of alcohol, communicated to it a bluish green color, as if the brain had undergone no alteration. This alcohol, on cooling, deposited a white matter, partly in flocks, partly in crystals. There remained a greyish substance, which the alcohol had not dissolved, and which resembled albumen.

From these experiments we may conclude,

1. That the fatty portion of the brain had undergone no sensible change during the putrefaction of this organ. It preserved the property when dissolved in alcohol to give it a green color, and to precipitate, on cooling, in a crystalline form, and retaining all its properties.

2. That a part only of the albumen was destroyed by the fermentation; that from this decomposition a small quantity of ammonia resulted which dissolved another portion of the albumen, and some acetic acid rendered sensible by the addition of sulphuric acid.

3. That the osmazome was not decomposed, at least completely, since its presence was still recognised in the concentrated liquid.

We conceive that the albumen of the brain putrifies much more speedily, and undergoes a more complete alteration, when it is in contact with the air than when it is confined in a close vessel.

I do not know what the substance is which assumes a red color during the putrefaction of the brain. I thought at first that it was the substance which gives a green color to alcohol; but I gave up that opinion when I saw that the cerebral matter still communicated the same color to alcohol after its putrefaction.

The cerebellum of man, and the brain of herbivorous animals, being examined in the same manner, and with the same precautions, yielded the same results. I propose to continue these researches on the brain of other classes of animals.

### Sect. XI.—*Of the medulla elongata, and spinal marrow.*

The medulla elongata and spinal marrow are of the same nature as the brain; but they contain much more fatty matter, and less albumen, osmazome, and water. Hence the reason why the spinal marrow has greater consistence than the brain.

The spinal marrow communicates to alcohol, when boiled in it, a blue color, as the brain does. It contains, likewise, superphosphate of of potash. The portion insoluble in alcohol is of the same nature as that of the brain; that is to say, albumen. The fatty matter contains phosphorus, like that of the brain.

*Of the nerves.*

The nerves are likewise of the same nature as the brain; but they contain much less fatty matter, and green coloring matter, and much more albumen. They contain, besides, common fat, which separates from them when treated with boiling alcohol, and deposites itself at the bottom of that liquid.

The nerves, deprived as much as possible of their fatty matter by means of alcohol, become semitransparent. Treated for a long time in that state with boiling water, they do not dissolve; but become white, opake, and swell up, obviously in consequence of absorbing moisture. The water in which they were boiled holds in solution a small quantity of matter; for the infusion of nutgalls forms a precipitate in it, and the solution properly evaporated yields a little jelly, derived, probably, from the cellular texture which binds the nervous fibres together.

After having been treated with alcohol and with water, the nerve dissolves almost completely in caustic potash. Only a few flocks remain, not amounting to the hundredth part of the mass employed. No ammonia is produced during the solution.

The solution of nerve in alkali is precipitated by acids, and the precipitate, as well as the liquid from which it fell, assumes a purple color.

Nerve preserved in water undergoes little alteration. The water, however, after a few days, assumes the odor of semen very sensibly.

Nerve put into oxymuriatic acid contracts its dimensions. As it is chiefly the envelope of the nerves which undergoes this change, the nervous substance issues from its case, and each of the fibres which compose it separates from those in its neighbourhood; so that the nerve looks like a hair-pencil with its extremities diverging. In this situation the substance of the nerve assumes more consistence and whiteness, owing to the condensation and opacity which it requires. From this experiment, it would seem that oxymuriatic acid would furnish a good instrument for facilitating the study of the nerves and their envelope.

Is it possible, from the experiments to which the brain has been subjected, to determine the state in which each of the elements composing this organ exists in it? Is not the albumen united to a portion of phosphoric acid, and is not its consistence and opacity owing to this combination? Without affirming any thing on this head, I will say, that this substance appears to have acquired its state of semicoagulation from an acid; just as happens to the curdy part of sour milk; and that this coagulation is produced entirely by a fermentation, which commences, like that of milk, by being acid.

I next proposed to myself this question: Is the fatty matter in combination with the albumen and the osmazome? This seems to

 be be the case, at least with regard to the fatty matter and albumen; for when the matter of brain is triturated with water, and converted into a species of emulsion, if it be left at rest the albumen and fatty matter separate together, and the osmazome remains in solution in the liquid, together with a small portion of the albumen. At the same time, I acknowledge that it is possible that these two substances are only in the state of mixture, and that the albumen here performs the same office to the fatty matter that mucilage* does to the oils of emulsive seeds.—*Annales de Chimie*, vol. lxxxi. p. 82.

* I call mucilage, with all the chemists, the substance which holds the oil in suspension in the emulsion of almonds, though it be of a very different nature from gum.

